# Thyroid Stimulating Hormone (TSH) Is Associated With General and Abdominal Obesity: A Cohort Study in School-Aged Girls During Puberty in East China

**DOI:** 10.3389/fendo.2020.00620

**Published:** 2020-09-30

**Authors:** Yingying Wang, Xiaolian Dong, Chaowei Fu, Meifang Su, Feng Jiang, Dongli Xu, Rui Li, Junhua Qian, Na Wang, Yue Chen, Qingwu Jiang

**Affiliations:** ^1^Department of Epidemiology, School of Public Health, Fudan University, Shanghai, China; ^2^Key Laboratory of Public Health Safety of Ministry of Education, Shanghai, China; ^3^Department of Chronic Disease Control and Prevention, Deqing County Center for Disease Control and Prevention, Huzhou, China; ^4^Department of Chronic Disease Control and Prevention, Yuhuan City Center for Disease Control and Prevention, Taizhou, China; ^5^Department of Chronic Disease Control and Prevention, Minhang District Center for Disease Control and Prevention, Shanghai, China; ^6^Department of Chronic Disease Control and Prevention, Haimen City Center for Disease Control and Prevention, Nantong, China; ^7^School of Epidemiology and Public Health, Faculty of Medicine, University of Ottawa, Ottawa, ON, Canada

**Keywords:** thyroid stimulating hormone, general obesity, central obesity, school-aged girls, puberty, cohort study

## Abstract

**Objectives:** Although the association between thyroid stimulating hormone (TSH) and obesity in children has been investigated in several cross-sectional studies, no study evaluated this association among girls during puberty, which were in a key period closely related to the fluctuations of thyroid hormones and development of obesity. Therefore, we conducted a cohort study to investigate the association of general and abdominal obesity with TSH in girls during puberty.

**Setting and participants:** A cohort study of 481 school-aged girls during puberty was conducted in four regions in east China, with a baseline survey in 2017 and a follow-up survey in 2019.

**Outcome measures:** Anthropometric indexes including height, weight and waist circumference (WC) were measured, and body mass index (BMI) was then calculated. Blood samples were collected to determine TSH and free thyroxine (FT4).

**Results:** Of the 474 girls at baseline survey, the prevalences of BMI-based general obesity and WC-based abdominal obesity were 19.8% (94/474) and 21.7% (103/474), respectively. Compared with normal weight girls, the median serum TSH level was significantly higher in general obese girls (*P* = 0.037), but not in central obese girls (*P* = 0.173). Multiple logistic regression models indicated that those in the highest tertile of serum TSH level had a significantly higher risk of BMI-based overweight/obesity (OR = 1.83, 95% CI 1.01 to 3.32) compared with the lowest tertile. Analyses from 435 girls prospectively followed-up for 2 years revealed that those with general or central obesity also had higher follow-up TSH level (*P* = 0.004 and *P* = 0.008, respectively). The TSH level for girls with general obesity at baseline but normal weight at follow-up was 0.45 mU/L (95% CI 0.11 to 0.79) higher than those with normal weight at baseline and follow-up.

**Conclusions:** TSH was positively associated with both general and abdominal obesity among girls during puberty.

## Introduction

Overweight and obesity are defined as abnormal and excess fat accumulation due to an imbalance between calorie intake and expenditure. The prevalence of overweight and obesity among children and adolescents aged 5–19 years has increased dramatically from 4% in 1975 to over 18% in 2016 globally (1). Childhood obesity has been linked to increased risks of asthma (2), metabolic syndrome (3), cardiovascular diseases (CVD) (4) and premature death in adulthood (5). Meanwhile, overweight compared with normal weight children are more likely to be obese in adulthood (6). Obesity is related to multiple endocrine alterations including various hormones (7, 8). Identifying hormonal targets may help obesity prevention and control.

Thyroid hormones are primarily involved in metabolism and energy expenditure of individuals (9). Evidence suggests that thyroid dysfunction predispose to obesity, or *vice versa* (10). Overweight and obese children tend to have higher TSH levels (11). The association between adiposity and thyroid dysfunction may be modified by sex. Females are more likely to have thyroid abnormalities as compared with males in adults, but not in children aged 8–10 years (12). The synthesis and secretion of thyroid hormones are controlled by hypothalamic-pituitary-thyroid axis, which is usually reactivated during puberty (13). The incidence of thyroid disorder in females increases upon the onset of puberty, and decreases after menopause (14). On the other hand, women with earlier menarche experience a higher risk of obesity (15). Girls during puberty therefore, provide a good opportunity to investigate the fluctuations of thyroid hormones in association with development of obesity, but this issue cannot be addressed in cross-sectional studies (16, 17). In this longitudinal study conducted in east China, we explored the association of TSH with overweight or obesity among girls during puberty, and assessed the potential effect modification of covariates.

## Subjects and Methods

### Study Population

**Figure 1:**
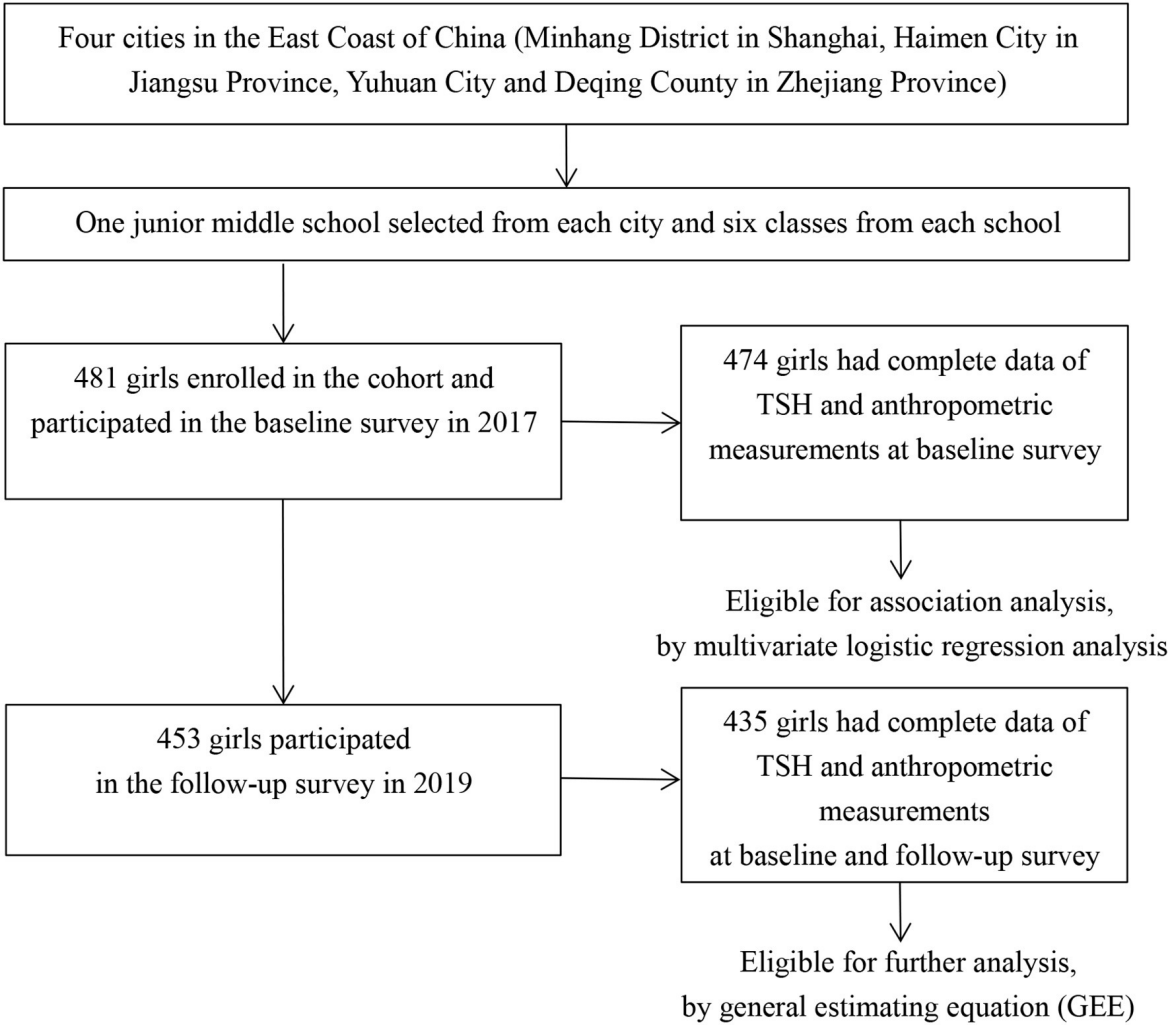
Flow chart for the study.

Four regions in East Coast of China (Minhang District in Shanghai, Haimen City in Jiangsu Province, Yuhuan City, and Deqing County in Zhejiang Province) were selected by purposive sampling. Previous studies have revealed an iodine-sufficient status along with distinguished iodized-salt consumption proportions among those sites (18–21). One junior middle school was randomly selected from each of the four regions and students were mainly local residents. A total of 481 girls from six classes of each school in grade 7 were enrolled into the cohort in October or November 2017 after excluding those with iodine supplement, thyroid disorder, pituitary abnormality or other conditions that affect thyroid hormone levels, and 474 of them completed both physical examinations and blood sample collections. Among 453 girls who participated in the follow-up investigation, 435 had complete data on thyroid function determinations and anthropometric measurements (Figure 1). Informed written consents were obtained from all the participants and their parents, and the study was approved by the ethical review board of the School of Public Health of Fudan University.

### Patient and Public Involvement

No patient involved.

### Information Collection and Variables Definition

Information on demographic and lifestyle factors were collected by a self-administrated questionnaire. Menarche (yes, no) and age at menarche were assessed. Important variables included monthly family income in Chinese Yuan (CNY) (≤3,000 CNY, >3,000 CNY), the highest education level for parents (junior high school or below, senior high school or above), daily bed time (≤8 h, >8 h) and daily activity time (≤1 h, >1 h).

### Anthropometric Measurements

Anthropometric measurements, including standing height (cm), weight (kg), and waist circumferences (cm) were taken by local health professionals according to a standard protocol. Height and weight were respectively measured to the nearest 0.1 cm and 0.1 kg with the subjects standing without shoes and wearing light clothing only. Waist circumference (WC) was measured to the nearest 0.1 cm at the midpoint between the lower rib and the upper iliac crest. Body mass index (BMI) was calculated as weight in kilograms divided by the square of height in meters (kg/m^2^).

### Thyroid Hormones Determination

Blood samples of ~5 mL were collected through antecubital vein puncture from each participant at both baseline and follow-up. Serum and blood clot were immediately centrifuged and kept at −80°C until analysis. Serum thyroid stimulating hormone (TSH) and free thyroid hormone (FT4) were determined by using an enzymatic cycling method.

### Urine and Salt Samples Collection and Iodine Content Determination

In order to distinguish the influences of home diet and school canteen diet on urine iodine concentration (UIC), each participant provided morning urine samples on a Monday and a Thursday, and we used the method of inductively coupled plasma mass spectrometry (ICP-MS) to determine the UICs. Weighted daily urine iodine output was calculated as follows: (1) daily urine iodine output (μg) = MCr×1000×UIC÷UCr, (22) (MCr: daily urine creatinine output, mmol/day, MCr = EXP(0.0102×H-0.6854; H: body height, cm; UIC: urine iodine concentration, μg/L; UCr: urine creatinine content, μmol/L); and (2) weighted daily urine iodine output (μg) = 2/7× daily urine iodine output on Monday + 5/7 × daily urine iodine output on Thursday.

Participants were also asked to bring a salt sample of more than 20 g from home and salt iodine was then measured using a national standard method with a proper quality control (GB/T 13025.7-2012) (23). Iodized-salt consumption status was grouped into two categories: non-iodized salt (<5 mg/kg) and iodized salt (≥5 mg/kg) (24). In our study, the proportions of iodized-salt consumption was 88.98, 95.45, 85.96, and 94.50% in Minhang, Haimen, Yuhuan, and Deqing, respectively.

### Statistical Analysis

Two patterns of obesity, general (peripheral) and central (abdominal) obesity, were evaluated by using body mass index (BMI) and waist circumference (WC), respectively (25). All participants were categorized into three groups of under/normal weight, overweight, and obesity according to the BMI growth reference values for Chinese children and adolescents aged 2–18 years suggested by Li et al. (26) and WC distribution of Chinese children and adolescents aged 7–18 years reported by Ji et al. (27). Participants were categorized into two groups (under/normal weight group, overweight/obesity) according to BMI due to small numbers for some subgroups.

Statistical description and cross-sectional analysis were performed based on data from 474 girls with complete information of serum TSH concentration and anthropometric measurements at baseline. Wilcoxon test and χ^2^ test were used to analyze continuous variables and categorical variables, respectively. Multiple logistic regression models were utilized to estimate the odds ratios (ORs) and 95% confidence intervals (95% CIs) for BMI- or WC-based overweight/obesity risks in relation to TSH levels (tertile 1, ≤1.53; mU/L, tertile 2, 1.54–2.37 mU/L; tertile 3, >2.37 mU/L). Major variables at baseline, including age, serum FT4 concentration, menarche or not, area with different proportions of iodized-salt consumption (≤90%, >90%) and weighted daily urine iodine output were taken into account. Stratified analysis was conducted according to age, menarche or not, area with different proportions of iodized-salt consumption, and their interactions with TSH levels were also assessed.

Girls who were lost to follow-up after 2 years or with missing data on TSH concentration or anthropometric measurements were excluded, leaving 435 girls with complete information for longitudinal data analysis. TSH levels at baseline or follow-up were compared between the under/normal weight and overweight/obesity groups. Four weight patterns were defined: (1) “B_N_F_N_”: under/normal weight at both baseline and follow-up; (2) “B_N_F_O_”: under/normal weight at baseline and overweight/obesity at follow-up; (3) “B_O_F_N_”: overweight/obesity at baseline and under/normal weight at follow-up; and (4) “B_O_F_O_”: overweight/obesity at both baseline and follow-up). General estimating equation (GEE) was used to evaluate the influences of the weight patterns, time and their interaction on serum TSH levels. The level of statistical significance was defined as α = 0.05 of two-side probability. All analyses were performed by using SPSS software for Windows (version 24.0, IBM Crop., Armonk, New York, USA). Figures were created by using GraphPad Prism software (version 7, California, USA).

## Results

The mean age for the 474 girls was 12.48 (±0.69) years at baseline. Of the participants, the prevalence was 19.83% for general overweight/obesity based on BMI, and was 21.73% for central overweight/obesity based on WC (Table 1). The prevalence of central overweight/obesity increased with age (*P* = 0.023). Girls who had experienced menarche had a higher risk of overweight/obesity compared with those who did not (*P* < 0.001). Generally overweight/obese girls had a higher serum TSH concentration than under/normal weight girls (median: 2.16 vs. 1.85 mU/L, *P* = 0.037), but serum FT4 levels show no significant difference between the two groups (*P* = 0.280). Centrally overweight/obese girls showed higher serum FT4 (*P* = 0.004) but similar TSH (*P* = 0.173) levels as compared with under/normal weight girls.

**Table 1:** Baseline characteristics of 474 girls with normal weight and overweight/obesity.

		**Classified based on body mass index (BMI)**			**Classified based on waist circumference (WC)**		
**Characteristics**	**Total**	**Under/normal Weight (*N* = 380)**	**Overweight (*N* = 74) or Obesity (*N* = 20)**	***P*-value**	**Under/normal Weight (*N* = 371)**	**Overweight (*N* = 69) or Obesity (*N* = 34)**	***P*-value**
**Thyroid stimulating hormone (TSH, mU/L)^a^**	1.90 (1.35–2.65)	1.85 (1.33–2.61)	2.16 (1.51–3.01)	0.037	1.85 (1.34–2.62)	2.14 (1.35–2.88)	0.173
**Free thyroid hormone (FT4, pmol/L)^a^**	14.83 (13.39–16.46)	14.79 (13.27–16.45)	15.24 (13.84–16.63)	0.280	14.68 (13.07–16.42)	15.46 (14.16–16.63)	0.004
**Weighted daily urine iodine output (μg)^a^**	90.57 (57.72–136.99)	89.17 (57.53–137.58)	98.89 (60.43–134.25)	0.817	89.61 (57.49–137.83)	95.70 (58.70–126.38)	0.899
**Age (*****N*****,%)**				0.994			0.023
11–12 years	217 (45.78)	174 (80.18)	43 (19.82)		180 (82.95)	37 (17.05)	
13–14 years	257 (54.22)	206 (80.16)	51 (19.84)		191 (74.32)	66 (25.68)	
**Menarche (*****N*****,%)**				<0.001			<0.001
No	176 (36.73)	159 (90.34)	17 (9.66)		156 (88.64)	20 (11.36)	
Yes	298 (62.87)	221 (74.16)	77 (25.84)		215 (72.15)	83 (27.85)	
**Area with different proportions of iodized-salt consumption (*****N*****,%)**				0.929			<0.001
≤ 90% (Minhang & Yuhuan)	244 (51.48)	196 (80.33)	48 (19.67)		209 (85.66)	35 (14.34)	
>90% (Haimen & Deqing)	230 (48.52)	184 (80.00)	46 (20.00)		162 (70.43)	68 (29.57)	
**Monthly family income (*****N*****,%)**				0.640			0.264
≤ 3,000 CNY	169 (35.65)	137 (81.07)	32 (18.93)		127 (75.15)	42 (24.85)	
>3,000 CNY	299 (63.08)	237 (79.26)	62 (20.74)		238 (79.60)	61 (20.40)	
**Parents' education (*****N*****,%)**				0.278			0.148
Junior high school or below	205 (43.25)	169 (82.44)	36 (17.56)		154 (75.12)	51 (24.88)	
Senior high school or above	264 (55.70)	207 (78.41)	57 (21.59)		213 (80.68)	51 (19.32)	
**Daily bedtime (*****N*****,%)**				0.743			0.781
≤ 8 h	251 (52.95)	202 (80.48)	49 (19.52)		197 (78.49)	54 (21.51)	
>8 h	217 (45.78)	172 (79.26)	45 (20.74)		168 (77.42)	49 (22.58)	
**Daily activity time (*****N*****,%)**				0.241			0.181
≤ 1 h	320 (67.51)	251 (78.44)	69 (21.56)		244 (76.25)	76 (23.75)	
>1 h	148 (31.22)	123 (83.11)	25 (16.89)		121 (81.76)	27 (18.24)	

a *Described as, median(P_25_–P_75_)*.

Multiple logistic regression analysis showed that girls in the highest tertile of serum TSH concentration had a significantly higher risk of BMI-based overweight/obesity compared with the lowest tertile after adjustment for potential confounders (OR = 1.83, 95% CI 1.01 to 3.32) (Table 2: Model 2), and that the adjusted OR 1.47 for both BMI-based and WC-based overweight/obesity risk in association with 1-SD increment in serum TSH (Table 2: Model 2).

**Table 2:** Associations of risk for overweight/obesity with TSH levels in 474 girls at baseline.

	**TSH at baseline (mU/L)^a^**			
	**Tertile1**	**Tertile2**	**Tertile3**	**Per 1-SD increase**
**BMI-BASED OVERWEIGHT/OBESITY**				
No of girls	158	160	156	–
No. of overweight/obesity	25	31	38	–
%	15.82	19.38	24.36	–
Model 1^b^	1.00	1.31 (0.73, 2.34)	1.76 (0.99, 3.12)	1.43 (1.08, 1.88)
Model 2^c^	1.00	1.33 (0.73, 2.42)	1.83 (1.01, 3.32)*	1.47 (1.10, 1.96)
Model 3^d^	1.00	1.31 (0.72, 2.42)	1.88 (1.02, 3.46)*	1.47 (1.10, 1.96)
**WC-BASED OVERWEIGHT/OBESITY**				
No of girls	158	160	156	–
No. of overweight/obesity	31	33	39	–
%	19.62	20.63	25.00	–
Model 1^b^	1.00	1.18 (0.67, 2.05)	1.57 (0.91, 2.72)	1.37 (1.04, 1.79)
Model 2^c^	1.00	1.26 (0.71, 2.24)	1.76 (0.99, 3.12)	1.47 (1.10, 1.94)
Model 3^d^	1.00	1.22 (0.68, 2.19)	1.70 (0.94, 3.06)	1.41 (1.06, 1.88)

a *Tertile1: ≤1.53 mU/L, Tertile2: 1.54–2.37 mU/L, Tertile3: >2.37 mU/L*.

b *Model 1: Adjusted for age alone*.

c *Model 2: Adjusted for age, serum FT4 levels, menarche or not, and area with different proportions of iodized-salt consumption*.

d *Model 3: Adjusted for age, serum FT4 levels, menarche or not, and weighted daily urine iodine output*.

**Figure 2:**
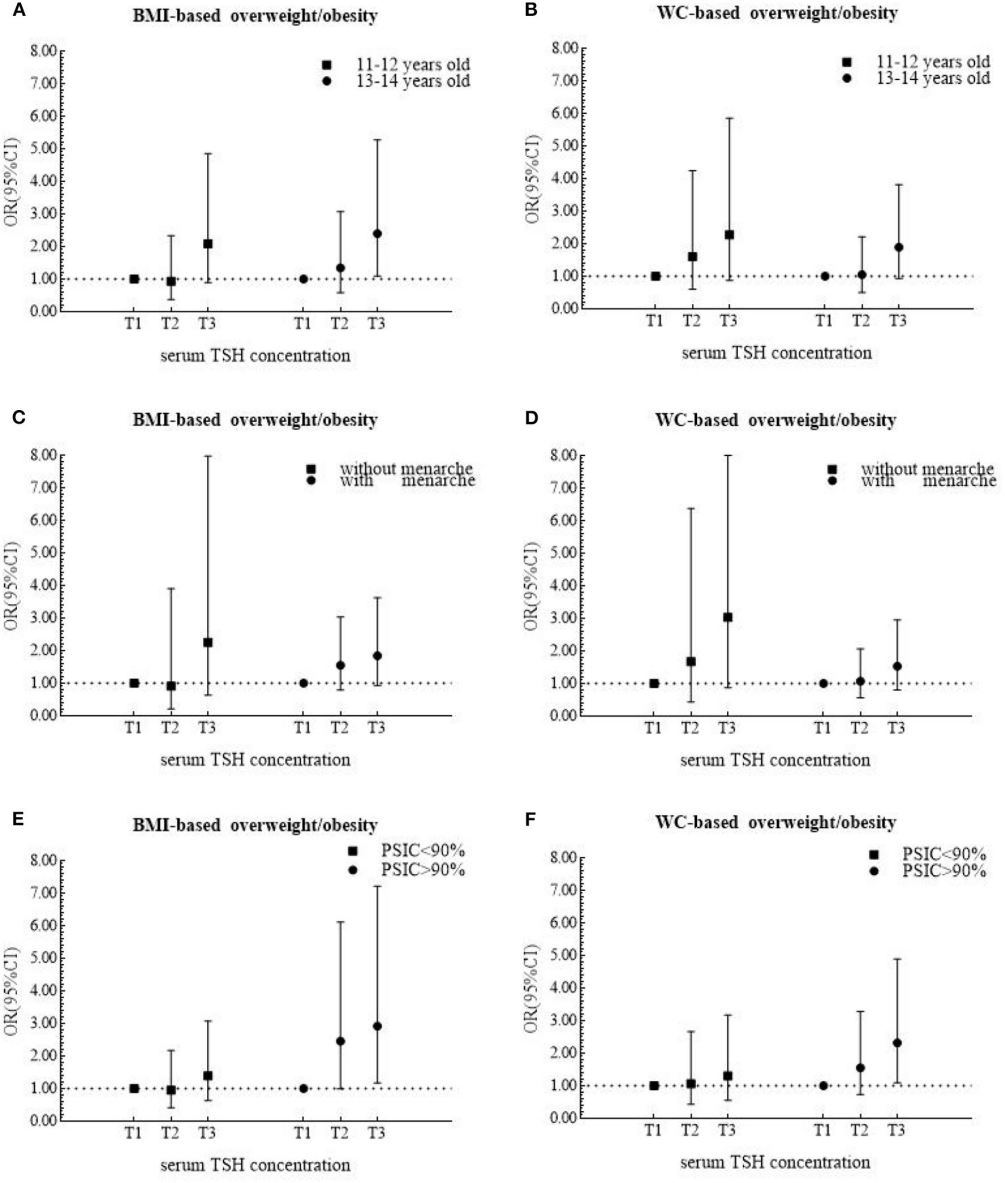
Odds ratios (ORs) and 95% confidence internals (95% CIs) for risk of overweight/obesity according to tertiles of serum TSH levels after being stratified by age, menarche and area in 474 girls.

In stratified analysis, serum TSH was positively related to the risks of BMI-based and WC-based overweight/obesity only among girls from area with the proportions of iodized-salt consumption more than 90% (BMI-based category: OR = 2.92, 95% CI 1.18 to 7.21; WC-based category: OR = 2.32, 95% CI 1.10 to 4.89) (Figures 2E,F). Among the older age group, the adjusted OR for BMI-based classification was 2.40 (95% CI 1.09 to 5.27) (Figure 2A).

**Figure 3:**
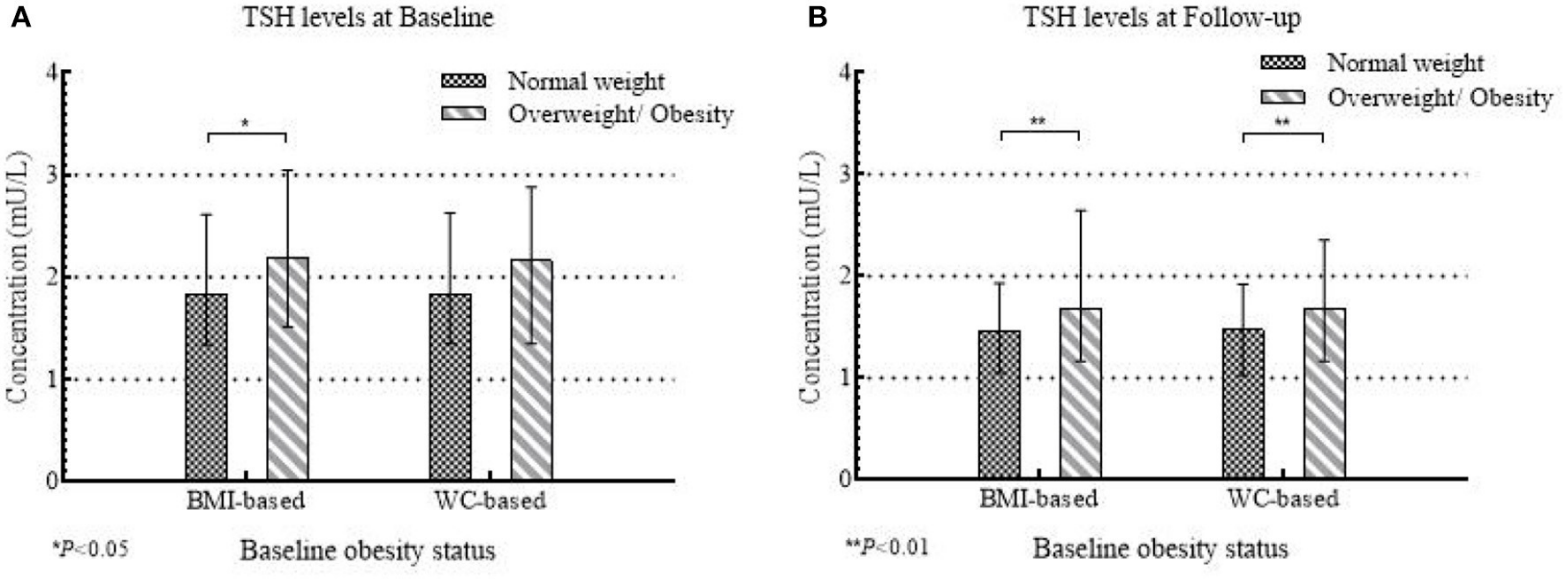
The concentrations of serum TSH at baseline and follow-up between normal weight and overweight/obese group according to baseline obesity status in 435 girls.

A total of 435 girls were followed up for 2 years, and the serum TSH concentration significantly decreased (*P* < 0.001) (Median: 1.90 mU/L at baseline and 1.50 mU/L at follow-up). The BMI-based overweight/obesity group maintained a higher level of serum TSH than the under/normal weight group both at baseline (*P* = 0.018) and follow-up (*P* = 0.004) (Figure 3A), but the difference was only observed for WC-based measure at follow-up (Figure 3B).

General estimating equation (GEE) indicated that there were main effects of BMI-based obesity development patterns (*P* = 0.016) and time (*P* < 0.001), but no interaction between them on the serum TSH levels (*P* > 0.05). Girls who experienced BMI-based overweight/obesity at baseline and then evolved to normal weight at follow-up (B_O_F_N_) had 0.42 mU/L (95% CI 0.07 to 0.77) higher serum TSH level than girls who kept normal weight (Table 3).

**Table 3:** Regression coefficients (βs) and 95% confidence internals (95% CIs) for serum TSH change over 2 years in 435 girls using general estimating equation (GEE).

	**BMI-based overweight/obesity**		**WC-based overweight/obesity**	
	**β(95%CI)^a^**	***P*-value**	**β(95%CI)^a^**	***P*-value**
**Obesity development patterns**		0.016		0.200
B_N_F_N_	0.00	–	0.00	
B_N_F_O_	0.50 (−0.03, 1.03)	0.063	0.09 (−0.19, 0.36)	0.544
B_O_F_N_	0.42 (0.07, 0.77)	0.020	0.26 (−0.20, 0.54)	0.069
B_O_F_O_	0.24 (−0.03, 0.50)	0.083	0.23 (−0.10, 0.55)	0.172
**Time**		<0.001		<0.001
Baseline	0.00	–	0.00	–
Follow-up	−0.46 (−0.55, −0.37)	<0.001	−0.46 (−0.55, −0.37)	<0.001

a *Adjusted for age, serum FT4 levels, menarche age (<11 years old, 11–12 years old, >12 years old), and area with different proportions of iodized-salt consumption*.

## Discussion

Our study indicated that in pubertal girls with a higher serum TSH level had a higher risk of overweight/obesity based on BMI or WC measures, and the association was more evident for the BMI measure. Previous studies demonstrated that TSH was positively related to BMI-based obesity in pediatric outpatients (11, 28), and WC-based obesity in adolescents (29, 30).

TSH, also known as thyrotropin, is produced by the anterior pituitary, regulated by the thyroid releasing hormone (TRH), and limited by the negative feedback from the thyroid hormones (THs) (31). THs primarily regulate basal metabolism, which constitutes approximately 66% of total daily energy expenditure (32). In addition, THs are involved in lipid and glucose metabolism with a mediator named sterol regulatory element-binding proteins (SREBP-2) (33, 34). Nader et al. found that increases in TSH levels within the reference range were associated with increases in insulin levels and homeostasis model assessment (HOMA) levels of insulin resistance(IR) (35), which may contribute to obesity in childhood (36). Insulin is a critical regulator of adipocyte biology, and promotes adipocyte triglyceride storage by some mechanisms, including transportation of glucose, differentiation of preadipocytes to adipocytes, and synthesis of triglyceride (lipogenesis) (37). Moreover, the positive relationship of TSH with triglyceride and non-HDL lipoproteins was indicated in a large population-based study among children and adolescents (38).

On the other hand, obesity may be a cause of elevated TSH levels (10). One plausible explanation is that adipose tissue secretes inflammatory cytokines into the general circulation, such as tumor necrosis factor (TNF-α) and interleukin (IL-1, IL-6) (39). These cytokines impede the expression of sodium iodine transporter mRNA and the activity of iodine uptake in human thyroid cells, which reduces the secretion of THs, and then leads a compensatory rise in TSH levels. Another hypothesis is that the production of leptin-mediated pro-thyrotropin-releasing hormone (pro-TRH) increases with weight gain (40). Leptin is predominantly released by adipocytes and stimulates TSH secretion by hypothalamic-pituitary axis (41). Non-synonymous mutations in thyroid stimulating hormone receptor (TSH-R) gene also plays a role in elevated TSH levels in relation to obesity (42).

In stratified analysis, the positive association between TSH and obesity was only observed in girls aged 13 to 14 years and from area with the proportions of iodized-salt consumption more than 90% (Haimen and Deqing), indicating that there may exist modification effect from age and iodine.

The strengths of our study include a cohort study enrolling school-aged girls, repeated measures of thyroid hormones and objectively anthropometric measurements following a standardized protocol collected at both baseline and follow-up. To our knowledge, rare studies have paid attention to girls around puberty for the relationship between thyroid function and adiposity based on two different evaluation criterion. However, there were several limitations. First, there is a lack of information on TPO-Ab (thyroid peroxidase antibody) that may be a better indicator of thyroid function. Moreover, our subjects were enrolled from sufficient-iodine and higher-economic areas in east China with a relatively small simple size, caution should be taken when extrapolating the results.

## Conclusion

In this prospective cohort study of girls during puberty, we found that TSH was positively associated with the risk of general or abdominal adiposity. Our findings emphasize the importance of preventing excess adiposity in girls with thyroid dysfunction.

## Strengths and limitations of this study

1. Main strengths include prospective cohort study design, targeting girls during puberty, repeated thyroid hormone measures and objectively anthropometric measurements following a standardized protocol collected at both baseline and follow-up.
2. Both central and abdominal adiposity were found to be association with increased levels of serum TSH.
3. Lack of information on TPO-Ab (thyroid peroxidase antibody), a better measure of thyroid function.

## Ethics Statement

The study was approved by the ethical review board of the School of Public Health of Fudan University (#2012-03-0350S). Written informed consent to participate in this study was provided by the participants' legal guardian/next of kin.

## Author Contributions

XD, MS, DX, JQ, NW, and QJ contributed to the study design. YW, CF, FJ, RL, and NW contributed to data acquisition and collection. YW, NW, and YC contributed to data analysis and interpretation, and drafted the manuscript. All authors contributed to the preparation of the final document, read, and approved the final manuscript.

## Conflict of Interest

The authors declare that the research was conducted in the absence of any commercial or financial relationships that could be construed as a potential conflict of interest.

## Abbreviations

TSH: thyroid stimulating hormone
TRH: thyroid releasing hormone
THs: thyroid hormones
FT4: free thyroxine
BMI: body mass index
WC: waist circumference
UIC: urine iodine concentration
ICP-MS: inductively coupled plasma mass spectrometry
GEE: general estimating equation.
